# Waist-to-height ratio, an optimal anthropometric indicator for metabolic dysfunction associated fatty liver disease in the Western Chinese male population

**DOI:** 10.1186/s12944-021-01568-9

**Published:** 2021-10-27

**Authors:** Jinwei Cai, Cuiting Lin, Shuiqing Lai, Yingshan Liu, Min Liang, Yingfen Qin, Xinghuan Liang, Aihua Tan, Yong Gao, Zheng Lu, Chunlei Wu, Shengzhu Huang, Xiaobo Yang, Haiying Zhang, Jian Kuang, Zengnan Mo

**Affiliations:** 1 The Second School of Clinical Medicine, Southern Medical University, Guangzhou, Guangdong China; 2 Department of Endocrinology, Guangdong Provincial People’s Hospital, Guangdong Academy of Medical Sciences, Guangzhou, Guangdong China; 3 Department of Endocrinology, The First Affiliated Hospital of Guangxi Medical University, Nanning, Guangxi Zhuang Autonomous Region China; 4 School of Pharmaceutical Sciences, Southern Medical University, Guangzhou, Guangdong China; 5 Department of Neurology, The First Affiliated Hospital of Guangxi Medical University, Nanning, Guangxi Zhuang Autonomous Region China; 6 Center for Genomic and Personalized Medicine, Guangxi Medical University, Nanning, Guangxi Zhuang Autonomous Region China; 7 Institute of Urology and Nephrology, The First Affiliated Hospital of Guangxi Medical University, Nanning, Guangxi Zhuang Autonomous Region China; 8 Guangxi Key Laboratory of Genomic and Personalized Medicine, Nanning, Guangxi Zhuang Autonomous Region China; 9 Guangxi Collaborative Innovation Center for Genomic and Personalized Medicine, Nanning, Guangxi Zhuang Autonomous Region China

**Keywords:** Waist-to-height ratio, Anthropometric indicator, Metabolic-dysfunction associated fatty liver disease, Lipid accumulation product, Abdominal volume index, Visceral fat, Non-alcoholic fatty liver disease

## Abstract

**Background:**

Non-alcoholic fatty liver disease (NAFLD) has been entitled as metabolic-dysfunction associated fatty liver disease (MAFLD). Therefore anthropometric indicators of adiposity may provide a non-invasive predictive and diagnostic tool for this disease. This study intended to validate and compare the MAFLD predictive and diagnostic capability of eight anthropometric indicators.

**Methods:**

The study involved a population-based retrospective cross-sectional design. The Fangchenggang area male health and examination survey (FAMHES) was used to collect data of eight anthropometric indicators, involving body mass index (BMI), waist-to-height ratio (WHtR), waist-hip ratio (WHR), body adiposity index (BAI), cardiometabolic index (CMI), lipid accumulation product (LAP), visceral adiposity index (VAI), and abdominal volume index (AVI). Receiver operating characteristics (ROC) curves and the respective areas under the curves (AUCs) were utilized to compare the diagnostic capacity of each indicator for MAFLD and to determine the optimal cutoff points. Binary logistic regression analysis was applied to identify the odds ratios (OR) with 95% confidence intervals (95% CI) for all anthropometric indicators and MAFLD. The Spearman rank correlation coefficients of anthropometric indicators, sex hormones, and MAFLD were also calculated.

**Results:**

All selected anthropometric indicators were significantly associated with MAFLD (*P* < 0.001), with an AUC above 0.79. LAP had the highest AUC [0.868 (95% CI, 0.853–0.883)], followed by WHtR [0.863 (95% CI, 0.848–0.879)] and AVI [0.859 (95% CI, 0.843–0.874)]. The cutoff values for WHtR, LAP and AVI were 0.49, 24.29, and 13.61, respectively. WHtR [OR 22.181 (95% CI, 16.216–30.340)] had the strongest association with MAFLD, regardless of potential confounders. Among all the anthropometric indicators, the strongest association was seen between LAP and sex hormones.

**Conclusion:**

All anthropometric indicators were associated with MAFLD. WHtR was identified as the strongest predictor of MAFLD in young Chinese males, followed by LAP and AVI. The strongest association was found between LAP and sex hormones.

**Supplementary Information:**

The online version contains supplementary material available at 10.1186/s12944-021-01568-9.

## Background

Non-alcoholic fatty liver disease (NAFLD) is highly prevalent and affects at least a quarter of the worldwide population [1, 2]. Habitually sedentary behavior, insufficient physical activity, uncontrolled high-calorie diet intake, and imbalanced nutritional expenditure can fuel NAFLD onset, progression, severity, and mortality [3]. Epidemiological surveillance data have shown that China has the highest incidence, prevalence, and annual NAFLD-related mortality in Asia [4]. Furthermore, the prevalence of NAFLD was substantially higher in males when compared to females in all Asian countries [4, 5]. Nevertheless, until very lately, NAFLD was not regarded as a growing public health concern in China [5].

Visceral fat accumulation has been reported as a major predictor and risk factor of NAFLD and linked with disease severity, particularly in patients on an excessively high-calorie diet [6–8]. Mechanistically, visceral adipose tissue may trigger lipotoxicity, severe insulin resistance (IR) and promote the release of proinflammatory and profibrogenic mediators. These factors eventually activate de novo hepatic lipogenesis, further exacerbating NAFLD [8, 9].

Recent studies [8, 10–12] demonstrated that various anthropometric indicators involving waist circumference (WC), body mass index (BMI), waist-to-height ratio (WHtR), waist-hip ratio (WHR), lipid accumulation product (LAP), visceral adiposity index (VAI), abdominal volume index (AVI), and body adiposity index (BAI) could be used to quantify visceral fat accumulation. However, studies have revealed a correlation between a strong ethnic heterogeneity of anthropometric indicators and NAFLD [5]. This may contribute to different liver fat distributions across different ethnicities. Moreover, most studies on NAFLD prevalence and anthropometric adiposity indicators included only Caucasian populations living in developed regions, hence limiting the generalizability of the research findings [5]. As a result, patients from some ethnic groups may receive an inaccurate clinical assessment. Therefore there is a need to validate further the predictive and diagnostic capacity of these anthropometric indicators amongst different ethnic populations living in various economic regions.

NAFLD is now being defined using a new set of diagnostic criteria and has been designated the novel nomenclature metabolic-dysfunction associated fatty liver disease (MAFLD) in 2020 [1, 13]. Lin et al. found that the novel MAFLD criteria were more accurate at diagnosing patients with fatty liver disease when compared with the NAFLD criteria [14]. However, to date, there is still no consensus on the most appropriate anthropometric indicator to quantify and predict NAFLD [6, 15], especially in the Chinese male population. Therefore, there is a need to identify appropriate indicators to predict and diagnose MAFLD while balancing both generalizability and eligibility.

To address this gap, this study was designed to evaluate the predictive power of eight anthropometric indicators (BMI, WHtR, WHR, BAI, LAP, VAI, AVI, and cardiometabolic index (CMI) [16]) and their optimal cutoff points for diagnosing MAFLD in Western Chinese male population.

## Materials and methods

### Study design and patient selection

**Fig. 1:**
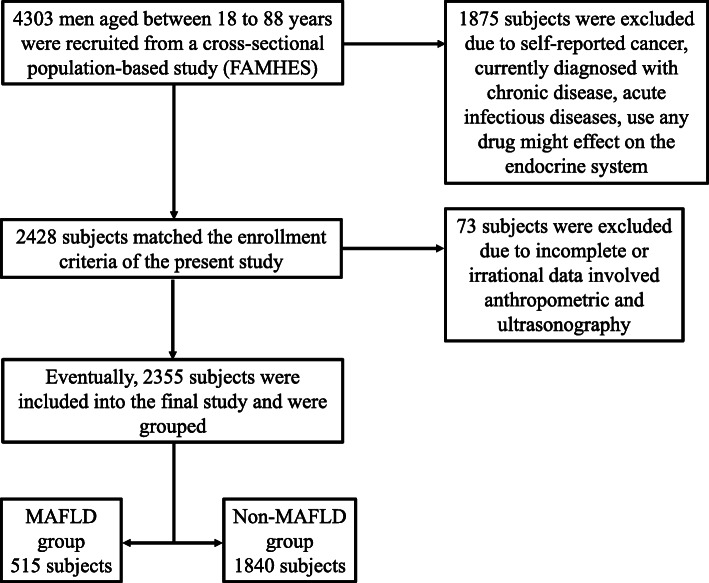
Flowchart of the study participants and exclusions

The current research was conducted using the Fangchenggang Area Male Health and Examination Survey (FAMHES), which was also used in previous studies [17–20]. FAMHES was a cross-sectional population-based study performed between September 2009 and December 2009 in the underdeveloped Guangxi Zhuang Autonomous Region of Western China. The study aimed to evaluate the impact of genetic as well as environmental factors involved in the development of age-associated conditions in non-hospitalized Chinese males. Local adult males (aged≥18) were asked to complete a regular survey after a physical examination at Fangchenggang First People’s Hospital. Their demographic data and physical examination results were recorded. Eventually, a total number of 4303 males aged between 18 to 88 years were non-consecutive enrolled and completed the survey. Patients who had cancer, chronic diseases, acute infectious diseases, and/or made use of any drugs that might affect the endocrine system were excluded from this study. Participants with incomplete anthropometric and liver ultrasound (US) records were also excluded (Fig. 1) [1].

### Ethical considerations

An informed consent from each participant was obtained. Ethical approval was given by the Medical Ethics Committee of the First Affiliated Hospital of Guangxi Medical University.

### Data collection

#### Questionnaire design and standardized protocol

Trained physicians used a standardized, structured, and detailed questionnaire for data collection. The collected data included demographic information (age, occupation, education, financial status, etc.), medical history (self-reported illness history and current medications), lifestyle hallmarks (physical activity, smoking, and alcohol consumption history), and family history of any chronic diseases. Anthropometric parameters were measured using a standardized protocol by trained personnel. Body shape measurements of waist, hip, and thigh circumferences were acquired with the participants dressed in thin clothes without shoes. A non-stretching tape was used to measure the waist and hip circumferences to the nearest 0.1 cm. The WC was determined at approximately halfway between the lowest margin of the last palpable rib and the top of the iliac. The hip circumference (HC) was defined as the perimeter surrounding the widest part of the buttocks at the axial plane [17, 21, 22]. Both height and weight were measured using digital scales to the nearest 0.1 cm and 0.1 kg, respectively. After at least a 5-min rest, the diastolic (DBP) and systolic (SBP) blood pressures were measured using a mercury sphygmomanometer or an automated device. Two measurements were acquired from the right arm with the participants in the seated position, and the readings were averaged. Participants were instructed to stop alcohol consumption, strenuous exercise, and smoking for at least 30 min prior to the assessment [17].

#### Biochemical measurements

After overnight fasting, blood specimens were acquired from the ulnar vein between 7 am and 9 am. The blood triglyceride (TG), total cholesterol (TC), high-density lipoprotein (HDL), low-density lipoprotein (LDL), serum alanine aminotransferase (ALT), uric acid (UA), fasting serum insulin, and fasting plasma glucose (FPG) were assayed. The homeostatic model assessment (HOMA)-IR, HOMA-β, and HOMA% Sensitivity (HOMA-IS) were subsequently calculated. Measurements of lipid parameters, fasting serum insulin, UA, and FPG were performed enzymatically applying a Dimension-RxL Chemistry Analyzer (Dade Behring, Newark, DE, USA) [17, 18]. An electrochemiluminescence immunoassay on COBAS 6000 system E601 (Elecsysmodule) immunoassay analyzers (Roche Diagnostics GmbH, Mannheim, Germany), was used to measure the blood values of sex hormones involving luteinizing hormone (LH), follicle-stimulating hormone (FSH), estradiol (E2), total testosterone (TT), and serum sex hormone-binding globulin (SHBG). Each sample was measured thrice and the interassay coefficient variations of FSH, LH, E2, TT, and SHBG were 4.3, 3.6, 3.4, 3.6 and 4.4%, respectively. All assays were conducted in accordance with the manufacturer’s instructions as previously described [18, 20, 23].

#### Candidate anthropometric predictors

All selected candidate anthropometric indicators were calculated by following standard equations:

[24]

$$ \mathrm{BMI}=\frac{\mathrm{Weight}\left(\mathrm{kg}\right)}{\mathrm{Height}{\left(\mathrm{m}\right)}^2} $$

[8]

$$ \mathrm{WHtR}=\frac{\mathrm{WC}\ \left(\mathrm{cm}\right)}{\mathrm{Height}\ \left(\mathrm{cm}\right)} $$

$$ \mathrm{WHR}=\frac{\mathrm{WC}\ \left(\mathrm{cm}\right)}{\mathrm{HC}\ \left(\mathrm{cm}\right)} $$

[25]

$$ \mathrm{BAI}=\left[\frac{\mathrm{HC}\ \left(\mathrm{cm}\right)}{\mathrm{Height}\ {\left(\mathrm{m}\right)}^{1.5}}\right]-18\kern0.5em $$

[16]

$$ \mathrm{CMI}=\mathrm{WHtR}\times \frac{\mathrm{TG}\left(\mathrm{mmol}/\mathrm{L}\right)}{\mathrm{HDL}\left(\mathrm{mmol}/\mathrm{L}\right)} $$

[26]

$$ \mathrm{LAP}=\left[\mathrm{WC}\ \left(\mathrm{cm}\right)-65\right]\times \mathrm{TG}\ \left(\mathrm{mmol}/\mathrm{L}\right)\ \mathrm{in}\ \mathrm{males} $$

[27]

$$ \mathrm{VAI}=\frac{{\mathrm{WC}}_{\left(\mathrm{cm}\right)}}{\left[39.68+\left(1.88\times \mathrm{BMI}\right)\right]}\times \left(\frac{{\mathrm{TG}}_{\left(\mathrm{mmol}/\mathrm{L}\right)}}{1.03}\right)\times \left(\frac{1.31}{{\mathrm{HDL}}_{\left(\mathrm{mmol}/\mathrm{L}\right)}}\right)\mathrm{in}\ \mathrm{males} $$

[28]

$$ \mathrm{AVI}=\frac{\left[2\times \mathrm{cm}{\left(\mathrm{WC}\right)}^2+0.7\times \mathrm{cm}{\left(\mathrm{WC}-\mathrm{HC}\right)}^2\right]}{1000} $$

#### Insulin resistance indices

IR were calculated by the standard formulas:

[29]

$$ \mathrm{HOMA}-\mathrm{IR}=\frac{\mathrm{fasting}\ {\mathrm{insulin}}_{\left(\upmu \mathrm{IU}/\mathrm{mL}\right)}\times {\mathrm{FPG}}_{\left(\mathrm{mmol}/\mathrm{L}\right)}}{22.5} $$

$$ \mathrm{HOMA}-\upbeta =\frac{20\times \mathrm{fasting}\ {\mathrm{insulin}}_{\left(\upmu \mathrm{IU}/\mathrm{mL}\right)}}{\left[{\mathrm{FPG}}_{\left(\mathrm{mmol}/\mathrm{L}\right)}-3.5\right]} $$

Or

[30]

$$ \mathrm{HOMA}-\mathrm{IR}=\frac{\left[{\mathrm{FPG}}_{\left(\mathrm{mg}/\mathrm{dL}\right)}\times \mathrm{fasting}\ {\mathrm{insulin}}_{\left(\upmu \mathrm{IU}/\mathrm{mL}\right)}\right]}{405} $$

$$ \mathrm{HOMA}-\upbeta =\frac{360\times \mathrm{fasting}\ {\mathrm{insulin}}_{\left(\upmu \mathrm{IU}/\mathrm{mL}\right)}}{\left[{\mathrm{FPG}}_{\left(\mathrm{mg}/\mathrm{dL}\right)}-63\right]} $$

HOMA-IS was calculated as reciprocal of HOMA-IR (1/HOMA-IR) [29].

#### Ultrasonography

The liver status of all participants, including echogenicity, posterior beam attenuation, size, contour, and structure, was evaluated by two experienced ultrasonographers utilizing a 5.0 MHz LOGIQ e portable ultrasound device (GE, Fairfield, CT, USA). The ultrasound diagnostic criteria of fatty liver were diffused liver enhanced near field echo, accompanying higher echoes in the hepatic parenchyma compared to the kidney; a blurred intrahepatic duct structure and an attenuated dirty liver far-field echo [20, 23, 31].

#### Definition of MAFLD

The novel positive diagnostic criteria of MAFLD can be applied regardless of the presence of other concomitant liver diseases or alcohol consumption. In present study, participants were diagnosed with MAFLD based upon evidence of hepatic stenosis according to histological, imaging or blood biomarkers and at least one of the major three clinical conditions, including type 2 diabetes mellitus states, overweight or obesity, and/or a metabolic disorder. The diagnosis of metabolic disorder required the occurrence of at least two metabolic risk abnormalities according to the MAFLD criteria described by Eslam et al. [1].

### Statistical analysis

Demographic and clinical characteristics of the participants were presented using means ± standard deviations for normally distributed variables and medians (interquartile range) for non-normally distributed continuous variables. Clinical characteristics and adipose tissue accumulation indicators were compared between groups (MAFLD and non-MAFLD) applying the Student’s *t*-test and the Wilcoxon-Mann-Whitney rank-sum tests. Receiver operating characteristic (ROC) curves were plotted, and the area under the ROC curves (AUCs) were calculated to compare the MAFLD diagnostic ability of all indicators. The indicator with the highest AUC was identified as the most valuable indicator. The maximum Youden index was utilized to define the optimal cutoff value and was calculated using the formula [Sensitivity + Specificity − 1] [32]. The Spearman rank correlation coefficients of anthropometric indicators, sex hormones, and MAFLD were calculated. Binary logistic regression models were constructed to explore correlations between anthropometric indicators (greater and less than the cutoff values presented in Table 3) and MAFLD. Potential confounding variables including age (continuous), blood glucose, blood pressure, plasma uric acid, lipid parameters, and sex hormone parameters were entered into the models in a stepwise manner. All statistical analyses were processed using the Statistical Package for the Social Sciences (SPSS) version 25.0 (IBM, Armonk, New York, USA). All the tests were two-tailed, and *P*-values below 0.05 were deemed statistical significance.

## Results

### Characteristics of the participants

A total number of 2355 participants were ultimately enrolled in this study. These were categorized into the MAFLD subgroup (*n* = 515) and the non-MAFLD subgroup (*n* = 1840) according to the novel MAFLD diagnostic criteria [1]. The prevalence of MAFLD among all participants was 21.87%. The characteristics of the participants are described in Table 1. The age, weight, WC, HC, SBP, DBP, ALT, TC, TG, LDL, UA, FPG, insulin, HOMA-IR, and HOMA-β were seen significantly higher in the MAFLD subgroup when compared with the non-MAFLD subgroup (all *P* < 0.001), while HDL, HOMA-IS, LH, E2, TT, and SHBG were significantly lower (all *P* < 0.001) among MAFLD sufferers. HOMA-IR, HOMA-β, and HOMA-IS were used to evaluate IR evaluation, insulin secretion and insulin sensitivity, respectively [33]. The current findings were consistent with previous results [20]. Meanwhile, height and FSH concentration did no differ significantly between the two groups.

**Table 1:** Baseline characteristics in study male population

Characteristics	Total (***n*** = 2355)	MAFLD (***n*** = 515)	Non-MAFLD (***n*** = 1840)	***P-*** Value
Age (years)	37.82 ± 11.10	39.84 ± 9.92	37.26 ± 11.35	< 0.001
Height (cm)	167.97 ± 5.54	167.98 ± 5.61	167.97 ± 5.53	0.967
Weight (kg)	65.87 ± 10.43	74.85 ± 9.58	63.36 ± 9.21	< 0.001
WC (cm)	80.87 ± 9.23	89.90 ± 6.75	78.34 ± 8.19	< 0.001
HC (cm)	91.51 ± 6.20	96.56 ± 5.59	90.10 ± 5.60	< 0.001
SBP (mmHg)	118.32 ± 15.48	123.53 ± 17.00	116.87 ± 14.70	< 0.001
DBP (mmHg)	77.00 ± 10.25	81.23 ± 11.34	75.82 ± 9.60	< 0.001
ALT (U/L)	39.00 (30.00–54.00)	54.00 (40.00–74.00)	36.00 (29.00–48.00)	< 0.001
**Lipid profile**				
TC (mmol/L)	5.71 ± 1.03	6.08 ± 1.03	5.60 ± 1.00	< 0.001
TG (mmol/L)	1.13 (0.78–1.75)	1.88 (1.31–2.79)	1.00 (0.71–1.48)	< 0.001
HDL (mmol/L)	1.40 ± 0.33	1.29 ± 0.40	1.44 ± 0.30	< 0.001
LDL (mmol/L)	2.97 ± 0.80	3.28 ± 0.78	2.88 ± 0.79	< 0.001
**Metabolic indices**				
UA (μmol/L)	378.46 ± 79.68	420.29 ± 87.04	366.76 ± 73.37	< 0.001
FPG (mmol/L)	5.20 (4.90–5.60)	5.40 (5.00–5.80)	5.10 (4.90–5.50)	< 0.001
Insulin (mU/L)	6.38 (4.27–9.67)	10.46 (7.57–15.22)	5.61 (3.85–8.19)	< 0.001
HOMA-IR	1.47 (0.97–2.31)	2.55 (1.80–3.75)	1.29 (0.87–1.90)	< 0.001
HOMA-IS	0.68 (0.43–1.03)	0.39 (0.27–0.56)	0.78 (0.53–1.14)	< 0.001
HOMA-β(%)	78.35 (49.82–118.33)	115.08 (79.45–164.45)	69.82 (45.86–104.74)	< 0.001
**Hormones**				
FSH (mIU/mL)	5.10 (3.58–7.53)	5.06 (3.63–7.53)	5.12 (3.56–7.54)	0.804
LH (mIU/mL)	5.18 (3.97–6.72)	4.89 (3.67–6.42)	5.27 (4.05–6.81)	< 0.001
Estradiol (pg/mL)	34.34 ± 10.13	32.58 ± 8.73	34.84 ± 10.44	< 0.001
Testosterone (ng/mL)	6.26 ± 1.93	4.92 ± 1.48	6.64 ± 1.87	< 0.001
SHBG (nmol/L)	38.35 (28.02–51.28)	25.84 (19.43–35.04)	41.97 (32.10–54.44)	< 0.001

*Abbreviations*: *MAFLD* Metabolic-dysfunction associated fatty liver disease, *WC* Waist circumference, *HC* HIP circumference, *SBP* Systolic blood pressure, *DBP* Diastolic blood pressure, *ALT* Alanine transaminase, *TC* Total cholesterol, *TG* Triglyceride, *HDL* High-density lipoprotein, *LDL* Low-density lipoprotein, *UA* Uric acid, *FPG* Fasting plasma glucose, *HOMA* Homeostatic model assessment, *FSH* Follicle stimulating hormone, *LH* Luteinizing hormone, *SHBG* Sex hormone-binding globulin. Numeric variables are described by (mean ± SD) or median (interquartile range). *P* < 0.05 was accepted statistically significant

### Anthropometric indicators differences between individuals with and without MAFLD

The anthropometric differences are summarized in Table 2. All anthropometric indicators in the MAFLD individuals were significantly higher when compared with the non-MAFLD individuals (all *P* < 0.001).

**Table 2:** Means ± standard deviations and medians (interquartile range) for the anthropometric indicators in individuals. (n = 2355)

	Total (***n*** = 2355)	MAFLD (***n*** = 515)	Non-MAFLD (***n*** = 1840)	***t-***Value ***Z-***Value	***P-***Value
BMI	23.32 ± 3.35	26.49 ± 2.82	22.44 ± 2.92	786.688	< 0.001
WHtR	0.48 ± 0.05	0.54 ± 0.04	0.46 ± 0.05	887.109	< 0.001
WHR	0.88 ± 0.06	0.93 ± 0.05	0.87 ± 0.05	583.939	< 0.001
BAI	24.08 ± 2.94	26.39 ± 2.65	23.43 ± 2.68	493.532	< 0.001
CMI	0.39 (0.24–0.72)	0.84 (0.54–1.28)	0.32 (0.21–0.55)	−22.183	< 0.001
LAP	17.09 (7.02–37.00)	45.24 (30.97–71.40)	12.09 (5.40–24.61)	−25.596	< 0.001
VAI	0.81 (0.50–1.43)	1.64 (1.02–2.60)	0.68 (0.45–1.13)	−21.308	< 0.001
AVI	13.35 ± 2.99	16.30 ± 2.45	12.52 ± 2.58	881.957	< 0.001

*Abbreviations*: *MAFLD* Metabolic-dysfunction associated fatty liver disease, *BMI* Body mass index, *WHtR* Waist to height ratio, *WHR* Waist to hip ratio, *BAI* Body adiposity index, *CMI* Cardiometabolic index, *LAP* Lipid accumulation product, *VAI* Visceral adiposity index, *AVI* Abdominal volume index. Numeric variables are described by (mean ± SD) or median (interquartile range). *P* < 0.05 was accepted statistically significant

### AUCs and cutoff MAFLD prediction points of all anthropometric indicators

**Fig. 2:**
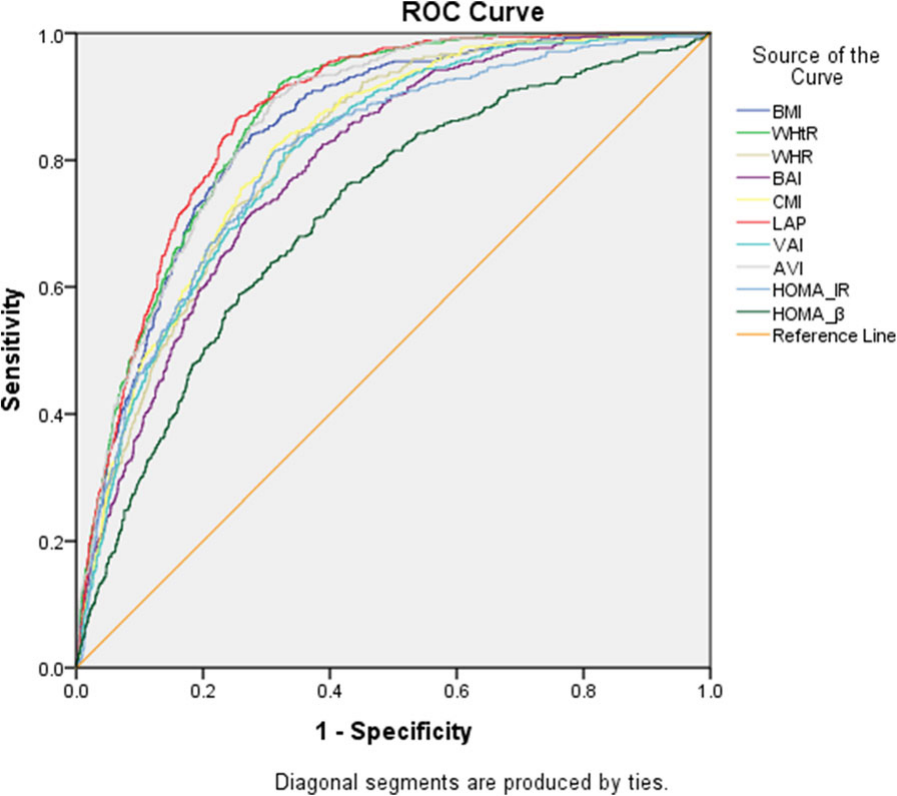
ROC-curves of different adipose tissue accumulation indicators and HOMA-index associate with MAFLD. Abbreviations: *MAFLD*: Metabolic-dysfunction associated fatty liver disease; *BMI*: Body; mass index; *WHtR*: Waist to height ratio; *WHR*: Waist to hip ratio; *BAI*: Body adiposity; index; *CMI*: Cardiometabolic index; *LAP*: Lipid accumulation product; *VAI*: Visceral; adiposity index; *AVI*: Abdominal volume index; *HOMA*: Homeostatic model assessment; *IR*: Insulin resistance

The ROC curves together with the optimal cutoff points for all indicators are displayed in Table 3 and Fig. 2. The AUC analysis showed that all eight selected anthropometric indicators had diagnostic value for MAFLD. However, LAP (AUC: 0.868 [95% CI, 0.853–0.883]) had the highest AUC and therefore the highest diagnostic value, closely followed by WHtR (AUC: 0.863 [0.848–0.879]), AVI (AUC: 0.859 [0.843–0.874]), BMI (AUC: 0.846 [0.829–0.864]), CMI (AUC: 0.819 [0.800–0.838]), WHR (AUC: 0.814 [0.796–0.833]), VAI (AUC: 0.807 [0.787–0.826]), and BAI (AUC: 0.790 [0.769–0.810]). The cutoff points at which the risk of MAFLD increased were 24.0 for BMI, 0.49 for WHtR, 0.89 for WHR, 24.97 for BAI, 0.48 for CMI, 24.29 for LAP, 0.94 for VAI, 13.61 for AVI, 1.69 for HOMA-IR, and 78.1 for HOMA-β (Table 3 and Fig. 2). When the determined cutoff points were applied to the ROCs, WHtR had the highest sensitivity (sens: 90.68%), whereas LAP had the highest specificity (spec: 74.73%).

**Table 3:** Cut-off points and AUCs (95% CI) of different anthropometric indicators and HOMA-index in predicting MAFLD (n = 2355)

Parameters	AUC (95% CI)	***P***-value	Cut-off point	Sensitivity (%)	Specificity (%)
BMI	0.846 (0.829–0.864)	< 0.001	24.00	84.08	72.39
WHtR	0.863 (0.848–0.879)	< 0.001	0.49	90.68	69.56
WHR	0.814 (0.796–0.833)	< 0.001	0.89	84.46	64.95
BAI	0.790 (0.769–0.810)	< 0.001	24.97	71.70	72.50
CMI	0.819 (0.800–0.838)	< 0.001	0.48	81.75	69.24
LAP	0.868 (0.853–0.883)	< 0.001	24.29	86.60	74.73
VAI	0.807 (0.787–0.826)	< 0.001	0.94	81.20	67.23
AVI	0.859 (0.843–0.874)	< 0.001	13.61	89.71	68.86
HOMA-IR	0.803 (0.783–0.824)	< 0.001	1.69	81.36	68.91
HOMA-β	0.715 (0.690–0.740)	< 0.001	78.10	76.31	57.18

*Abbreviations*: *MAFLD* Metabolic-dysfunction associated fatty liver disease, *BMI* Body mass index, *WHtR* Waist to height ratio, *WHR* Waist to hip ratio, *BAI* Body adiposity index, *CMI* Cardiometabolic index, *LAP* Lipid accumulation product, *VAI* Visceral adiposity index, *AVI* Abdominal volume index, *HOMA* Homeostatic model assessment, *AUC* Area under the ROC curves, *P* < 0.05 was accepted statistically significant

### Spearman rank correlation analysis of anthropometric indicators, sex hormones and MAFLD

The spearman rank correlation coefficients of anthropometric indicators with sex hormones in MAFLD individuals and both MAFLD and non-MAFLD groups combined are summarized in Table 4 and Supplementary Table S1. A statistically significant negative correlation was noted between all anthropometric indicators with SHBG and TT (all *P* < 0.001), for the combined group analysis. In contrast, a stable significant positive correlation was seen for all anthropometric indicators with HOMA-IR and HOMA-β (all *P* < 0.001). Amongst all anthropometric indicators, LAP had the strongest correlation with the sex hormones SHBG, E2, TT, and HOMA-IR. Further analysis for the MAFLD group revealed a broadly consistent correlation between the anthropometric indicators and sex hormones except for SHBG, whereby significant negative correlations with BMI, CMI, LAP, VAI, and AVI were noted. The strongest correlations were observed between LAP with TT and HOMA-IR, as well as VAI and SHBG.

**Table 4:** Spearman rank correlation analysis of anthropometric indicators and sex hormones in MAFLD Population (n = 515)

Variable	SHBG		FSH		LH		E2		TT		HOMA-IR		HOMA-β	
	*R*-value	*P*-value	*R*-value	*P*-value	*R*-value	*P*-value	*R*-value	*P*-value	*R*-value	*P*-value	*R*-value	*P*-value	*R*-value	*P*-value
BMI	−0.173	< 0.001	−0.017	0.707	−0.052	0.243	0.133	0.003	−0.212	< 0.001	0.420	< 0.001	0.332	< 0.001
WHtR	−0.083	0.061	0.132	0.003	0.041	0.358	0.100	0.023	− 0.214	< 0.001	0.401	< 0.001	0.294	< 0.001
WHR	−0.045	0.307	0.209	< 0.001	0.105	0.018	−0.022	0.612	−0.187	< 0.001	0.302	< 0.001	0.164	< 0.001
BAI	−0.070	0.112	−0.021	0.631	−0.049	0.269	0.120	0.006	−0.128	0.004	0.246	< 0.001	0.244	< 0.001
CMI	−0.264	< 0.001	−0.043	0.327	−0.028	0.522	−0.062	0.159	−0.279	< 0.001	0.334	< 0.001	0.217	< 0.001
LAP	−0.274	< 0.001	0.007	0.869	−0.033	0.455	−0.064	0.147	−0.324	< 0.001	0.431	< 0.001	0.247	< 0.001
VAI	−0.278	< 0.001	− 0.072	0.101	− 0.049	0.265	− 0.055	0.211	− 0.273	< 0.001	0.323	< 0.001	0.210	< 0.001
AVI	−0.119	0.007	0.100	0.023	0.002	0.958	0.127	0.004	−0.223	< 0.001	0.423	< 0.001	0.306	< 0.001

*Abbreviations*: *MAFLD* Metabolic-dysfunction associated fatty liver disease, *BMI* Body mass index, *WHtR* Waist to height ratio, *WHR* Waist to hip ratio, *BAI* Body adiposity index, *CMI* Cardiometabolic index, *LAP* Lipid accumulation product, *VAI* Visceral adiposity index, *AVI* Abdominal volume index, *SHBG* Sex hormone-binding globulin, *FSH* Follicle stimulating hormone, *LH* Luteinizing hormone, *E2* Estradiol, *TT* Testosterone, *HOMA* Homeostatic model assessment. *P* < 0.05 was accepted statistically significant

### Binary logistic regression analysis of anthropometric indicators in predicting MAFLD

The binary logistic regression analysis explored that all anthropometric indicators significantly improved the diagnostic predictivity for MAFLD (all *P* < 0.001) even after correcting for potential confounding variables. However, WHtR had the strongest association with MAFLD [OR 22.181 (95% CI, 16.216–30.340)], followed by AVI, LAP, BMI, CMI, WHR, HOMA-IR, VAI, BAI, and HOMA-β. After adjustment for potential confounding variables involving age (continuous), blood glucose, blood pressure, plasma uric acid, lipid parameters, and sex hormone parameters separately (model I, II, and III), the ORs of selected indicators decreased dramatically for all models. Yet, WHtR consistently exhibited the highest OR, suggesting that WHtR is the best predictor for MAFLD for all models. Although in the ROC curve analysis, LAP had the highest AUC, it was very closely followed by WHtR, which further confirming the strongest predictive and diagnostic power of WHtR. The outcomes of the multivariate binary logistic regression models analysis suggest that advanced age, hypertension, hyperuricemia, hypertriglyceridemia, and FSH were risk factors, while high values of LH, TT, and SHBG were protective factors for MAFLD. The results were generally consistent with the findings of previous studies [20, 31, 34–36]. In model II, HDL was a protective factor, and diabetes was a risk factor. Nevertheless, after adjusting for sex hormones and SHBG (model III), HDL was no longer a statistically significant protective factor for MAFLD in the CMI, LAP, and VAI models. Similarly, hypertriglyceridemia was no longer a statistically significant risk factor in the CMI and LAP models. Elevated plasma glucose was no longer identified as a significant factor for MAFLD in all anthropometric models except BAI. The details of the binary logistic regression analysis are available in Table 5 and Supplementary Table S2.

**Table 5:** Binary logistic regression analysis of anthropometric indicators and HOMA-index in predicting MAFLD (*n* = 2355)

Variable	Non-adjusted		Model I		Model II		Model III	
	OR(95% CI)	*P*-value	OR(95% CI)	*P*-value	OR(95% CI)	*P*-value	OR(95% CI)	*P*-value
BMI	13.722 (10.620–17.729)	< 0.001	12.768 (9.866–16.525)	< 0.001	7.431 (5.635–9.800)	< 0.001	5.019 (3.751–6.715)	< 0.001
WHtR	22.181 (16.216–30.340)	< 0.001	21.441 (15.575–29.516)	< 0.001	12.454 (8.880–17.466)	< 0.001	7.795 (5.492–11.064)	< 0.001
WHR	10.050 (7.773–12.994)	< 0.001	9.583 (7.335–12.521)	< 0.001	5.468 (4.107–7.280)	< 0.001	3.924 (2.912–5.287)	< 0.001
BAI	6.663 (5.362–8.280)	< 0.001	6.248 (5.013–7.786)	< 0.001	4.014 (3.159–5.099)	< 0.001	2.922 (2.267–3.767)	< 0.001
CMI	10.081 (7.894–12.874)	< 0.001	9.459 (7.394–12.101)	< 0.001	4.969 (3.589–6.880)	< 0.001	3.288 (2.339–4.621)	< 0.001
LAP	16.856 (12.825–22.155)	< 0.001	15.734 (11.952–20.714)	< 0.001	9.029 (6.560–12.426)	< 0.001	6.077 (4.345–8.501)	< 0.001
VAI	8.818 (6.927–11.226)	< 0.001	8.331 (6.532–10.627)	< 0.001	3.933 (2.855–5.417)	< 0.001	2.685 (1.917–3.759)	< 0.001
AVI	19.275 (14.266–26.041)	< 0.001	18.107 (13.352–24.555)	< 0.001	10.359 (7.498–14.311)	< 0.001	6.537 (4.676–9.138)	< 0.001
HOMA-IR	9.675 (7.590–12.334)	< 0.001	9.761 (7.634–12.481)	< 0.001	6.149 (4.689–8.064)	< 0.001	4.384 (3.298–5.828)	< 0.001
HOMA-β	4.281 (3.425–5.352)	< 0.001	4.710 (3.748–5.918)	< 0.001	3.594 (2.768–4.666)	< 0.001	2.792 (2.110–3.694)	< 0.001

*Abbreviations*: *MAFLD* Metabolic-dysfunction associated fatty liver disease, *BMI* Body mass index, *WHtR* Waist to height ratio, *WHR* Waist to hip ratio, *BAI* Body adiposity index, *CMI* Cardiometabolic index, *LAP* Lipid accumulation product, *VAI* Visceral adiposity index, *AVI* Abdominal volume index, *HOMA* Homeostatic model assessment, *OR* Odds ratio, *CI* Confidence interval. *P* < 0.05 was accepted statistically significant

Model I:Adjusted for age

Model II:Adjusted for age, blood glucose, blood pressure, plasma uric acid, lipid parameters (TC, TG, HDL-C, LDL-C)

Model III:Adjusted for age, blood glucose, blood pressure, plasma uric acid, lipid profile and sex hormone parameters (FSH, LH, E2, TT, SHBG)

## Discussion

The prevalence of MAFLD in FAMHES conducted in the Guangxi area turned out to be 21.87%, significantly lower than the national overall NAFLD prevalence of China over the last two decades [29.6% (95% CI, 28.2–31.0%)] [4]. This counterintuitive outcome reflects the complex and the potential higher incidence of NAFLD modifiable risk factors in industrialized regions when compared with urban areas. Nonetheless, most studies performed during the past decade on NAFLD prevalence have mainly been conducted in developed regions, potentially limiting the generalizability of these studies to less developed regions [5].

Numerous studies have demonstrated that anthropometric indicators of visceral fat can improve the predictive performance of current chronic diseases models, including central adiposity, diabetes mellitus, cardiometabolic diseases, left ventricular hypertrophy, hyperuricemia, and metabolic syndrome [7, 8]. Nevertheless, hitherto, remarkably few studies have adequately assessed the diagnostic capabilities of anthropometric indicators and sex hormones in MAFLD.

The prevalence of NAFLD was found to be proportional to the increase in BMI [34]. BMI has been used as a surrogate index for visceral fat and was widely used to evaluate NAFLD [6, 37]. However, its validity as an appropriate indicator of obesity has been criticized due to its inability to distinguish between lean body mass and fat mass and characterize regional adipose distribution. Furthermore, it does not account for racial or ethnic heterogeneity and varies by gender despite comparable body fat proportions [9, 12]. Ju et al. [38] and Zheng et al. [39] obtained an AUC of 0.760 (95% CI, 0.747–0.773) for BMI in males and 0.854 (95% CI, 0.78–0.93) in general population respectively in a cross-sectional study. The findings of the current study concur with previous studies [8, 40, 41]. BMI still had a reliable predictive value and satisfactory sensitivity and specificity for MAFLD, yet its predictive performance absented striking predominance when compared with the other aforementioned indices.

Research on the use of WHR and WHtR as anthropometric indicators to predict NAFLD is still limited and inconclusive. Zheng et al. [39] showed that WHR with a cutoff point of 0.89 (sens: 99%, spec: 66%) had a higher diagnostic value [AUC: 0.916 (95% CI, 0.86–0.97)] for NAFLD when compared with WHtR [AUC: 0.878 (95% CI, 0.82–0.94)], with a cutoff point of 0.49 (sens: 96%, spec: 64%). These findings are consistent with the present study whereby the cutoff points of 0.89 for WHR and 0.49 for WHtR, respectively. Conversely, WHtR had the strongest prediction performance and optimal diagnostic capability for MAFLD in current study. A similar finding was also noted by the study of Motamed et al. [7], whereby WHtR had the strongest association with NAFLD. Procino et al. [41] compared the performance of various indicators using a two-step hybrid method for NAFLD diagnosis, and WHtR was identified as the optimal indicator, eventually reducing the demand for abdominal US. There are diverse results had been reported [7, 41, 42]. Notably, the study by Zhang et al. [39] was conducted on a population with a higher proportion of young males with a mean age of 37.32 (±10.19) years, while the mean age in this study was 37.82 (±11.10) years. Therefore, both studies had the same cutoff points for WHR and WHtR suggesting that the results are more useful at predicting MAFLD in relatively young Chinese males.

LAP and VAI were defined as novel sex-specific indices with high accuracy and efficiency in identifying visceral obesity and cardiovascular risk assessment, particularly in older men [27, 43]. Zhang et al. [40] demonstrated that LAP is a practical, valuable tool in the prediction of NAFLD, especially in the elderly population (age ≥ 65 years). Another large cross-sectional study by Dai et al. [44] from China highlighted that LAP showed high accuracy for discriminating NAFLD. Lin et al. [8] demonstrated that LAP was more prominently correlated with NAFLD in females than in males. However, in current study, the optimal LAP cutoff point for predicting NAFLD was 24.29, substantially lower when compared with the previously reported cutoff points of 36.15 [40] and 30.05 [44]. This reported discrepancy could be attributed to variations in age, economic and social disparities, and the high levels of economic inequality in urbanized regions. The diagnostic effectiveness of VAI in NAFLD individuals remains controversial. Several studies claimed that VAI was not associated with liver histology [45–47], while Patta et al. [48] identified a significant correlation between VAI and hepatic fibrosis in NAFLD patients. According to the present study, LAP had the highest diagnostic value for MAFLD, whereas VAI ranked lower when compared with other indicators. Similar to previously published studies, LAP also had the strongest correlation with sex hormones [8, 49]. Nonetheless, studies evaluating the association between anthropometric indicators and sex hormones are currently limited, highlighting the need for further research.

Another intriguing finding in the current study was that AVI, a previously neglected indicator, was identified as a strong predictor for MAFLD, achieving the second-highest OR and third-highest AUC. Lin et al. [8] assessed the association between AVI and BAI with NAFLD in both male and female Taiwanese adults. The AVI and BAI AUCs were 0.700 and 0.616 respectively in males. When combined with US, Procino et al. [41] identified AVI as the best indicator for NAFLD. Verma et al. [11] implicated that BAI was the most specific and sensitive index for predicting overweight and obesity in females, while WHtR was the most reliable and sensitive surrogate indicator of obesity in males when compared with WC, BMI, and WHR. Heretofore, the role of CMI as a predictor for NAFLD has not been evaluated. Kendel Jovanovic et al. [50] reported that a substantial decrease in CMI was correlated with a significant decrease in total and visceral fat mass. When compared with other indicators, AVI, BAI, and CMI were not identified as significant indicators for NAFLD in previous similar studies [8]. As an unexpected harvest, the ROC curve analysis in current study empowered AVI, BAI, and CMI with new vision for their outstanding predictive and diagnostic performance of MAFLD with an AUC above 0.79 for all indicators. Other indicators of adipose accumulation used in prior studies, such as body shape index (ABSI) [40] and body roundness index (BRI) [8], were not evaluated in this study due to the limitation of their cumbersome formulas and the already proven poor diagnostic power for NAFLD [7, 40, 41].

### Comparisons with other studies and what does the current work add to the existing knowledge

The present study differs from previously published work. It evaluated the prognostic value of anthropometric indicators for the MAFLD population as opposed to the NAFLD population, bringing in a new perspectives. Additionally, this study evaluated for the first time the role of the hormones and anthropometric indicators in the initiation and progression of MAFLD. Compare to the similar studies published recently [8, 15, 40, 41], the controversial results in current study indicate that ethnicity, age, regional economic variations, and social inequalities within the urbanized populations can influence the value of anthropometric indicators for detecting MAFLD, highlighting the need to take into account population variations when applying prediction models.

### Study strengths and limitations

This is the first, large-scale, single-center retrospective cross-sectional study comparing the predictive capability of eight anthropometric indicators of visceral adiposity in MAFLD individuals within the Chinese male population. The optimal diagnostic predictors for MAFLD were identified for a unique population. The correlation of anthropometric indicators, sex hormones, and MAFLD were first described. Nevertheless, the study has some limitations that have to be acknowledged. The FAMHES study did not include female participants, and therefore, it could not compare the efficacy of these indicators between gender. The study performed as a retrospective cross-sectional design, therefore it could not identify other potential causal relationships and might be limited to predict the current MAFLD risk. Additionally, the study only included the ethnic-specific Western Chinese population, potentially limiting the generalizability of the results to other populations. Finally, the limited available studies evaluating the association between anthropometric indicators and liver fat deposits were generally conducted amongst the NAFLD population and not MAFLD. This made it difficult to compare the current findings with previously published literature.

## Conclusion

To summarize, this study detailed the significant impact of MAFLD prevalence in Western China. It simultaneously evaluated the effect of ethnic heterogeneity and regional variations in the anthropometric indicators and their associations with MAFLD. All eight anthropometric indicators illustrated the superior diagnostic value of MAFLD. Principally, WHtR was identified as the most powerful diagnostic predictor for young males with MAFLD in Western China, followed by LAP and AVI. The strongest association was found between LAP and sex hormones. The present findings can be used to develop valid and reliable clinical models for the early prediction and diagnosis of MAFLD in high-risk underdeveloped Chinese communities, facilitating the implementation of early and effective curative interventions.

## Supplementary Information

**Additional file 1: Table S1.** Spearman rank correlation analysis of anthropometric indicators and sex hormones in study male population (*n* = 2355).

**Additional file 2: Supplementary Table S2.** Binary logistic regression analysis of anthropometric indicators in predicting MAFLD.

## Data Availability

All data used in this study are available from the corresponding author.

## Abbreviations

NAFLD: Non-alcoholic fatty liver disease
MAFLD: Metabolic-dysfunction associated fatty liver disease
FAMHES: Fangchenggang Area Male Health and Examination Survey
BMI: Body mass index
WHtR: Waist-to-height ratio
WHR: Waist-hip ratio
BAI: Body adiposity index
CMI: Cardiometabolic index
LAP: Lipid accumulation product
VAI: Visceral adiposity index
AVI: Abdominal volume index
ROC: Receiver operating characteristic curve
AUC: Area under the ROC curves
ORs: Odds ratios
CI: Confidence interval
WC: Waist circumference
BRI: Body roundness index
ABSI: Body shape index
HC: Hip circumference
SBP: Systolic blood pressure
DBP: Diastolic blood pressure
TG: Triglyceride
TC: Total cholesterol
HDL: High-density lipoprotein
LDL: Low-density lipoprotein
ALT: Alanine aminotransferase
UA: Uric acid
FPG: Fasting plasma glucose
FSH: Follicle stimulating hormone
LH: Luteinizing hormone
TT: Total testosterone
E2: Estradiol
SHBG: Serum sex hormone-binding globulin
IR: Insulin resistance
HOMA: Homeostatic model assessment
HOMA-IS: HOMA% Sensitivity
US: Ultrasound
Sens: Sensitivity
Spec: Specificity
