# Supplementary material for: Waist-to-height ratio, an optimal anthropometric indicator for metabolic dysfunction associated fatty liver disease in the Western Chinese male population
Source: Lipids Health Dis. 2021 Oct 27;20:145. doi: 10.1186/s12944-021-01568-9 (PMC8549212; doi:10.1186/s12944-021-01568-9)
Supplement: Supplementary file 1 — Additional file 1: Table S1. Spearman rank correlation analysis of anthropometric indicators and sex hormones in study male population (n = 2355). [file 12944_2021_1568_MOESM1_ESM.docx]

**Supplementary Table S1:** **Spearman rank correlation analysis of anthropometric indicators and sex hormones** **in study male population (n=2355).**

| Variable | SHBG | | FSH | | LH | | E2 | | TT | | HOMA-IR | | HOMA-β | |
| --- | --- | --- | --- | --- | --- | --- | --- | --- | --- | --- | --- | --- | --- | --- |
|  | *R*-value | *P*-value | *R*-value | *P*-value | *R*-value | *P*-value | *R*-value | *P*-value | *R*-value | *P*-value | *R*-value | *P*-value | *R*-value | *P*-value |
| BMI | -0.476 | <0.001 | 0.520 | 0.012 | -0.033 | 0.109 | -0.024 | 0.236 | -0.468 | <0.001 | 0.560 | <0.001 | 0.444 | <0.001 |
| WHtR | -0.440 | <0.001 | 0.114 | <0.001 | 0.010 | 0.637 | -0.057 | 0.006 | -0.488 | <0.001 | 0.574 | <0.001 | 0.445 | <0.001 |
| WHR | -0.336 | <0.001 | 0.145 | 0.012 | 0.039 | 0.061 | -0.081 | <0.001 | -0.408 | <0.001 | 0.480 | <0.001 | 0.350 | <0.001 |
| BAI | -0.377 | <0.001 | 0.061 | 0.003 | -0.019 | 0.361 | -0.034 | 0.096 | -0.409 | <0.001 | 0.465 | <0.001 | 0.370 | <0.001 |
| CMI | -0.469 | <0.001 | -0.016 | 0.440 | -0.056 | 0.006 | -0.083 | <0.001 | -0.451 | <0.001 | 0.531 | <0.001 | 0.419 | <0.001 |
| LAP | -0.505 | <0.001 | 0.044 | 0.033 | -0.038 | 0.065 | -0.105 | <0.001 | -0.514 | <0.001 | 0.589 | <0.001 | 0.454 | <0.001 |
| VAI | -0.470 | <0.001 | -0.037 | 0.073 | -0.066 | 0.001 | -0.071 | 0.001 | -0.433 | <0.001 | 0.515 | <0.001 | 0.412 | <0.001 |
| AVI | -0.458 | <0.001 | 0.087 | <0.001 | -0.002 | 0.915 | -0.032 | 0.116 | -0.476 | <0.001 | 0.579 | <0.001 | 0.462 | <0.001 |

**Abbreviations:** *MAFLD*: Metabolic-dysfunction associated fatty liver disease; *BMI*: Body mass index; *WHtR*: Waist to height ratio; *WHR*: Waist to hip ratio; *BAI*: Body adiposity index; *CMI*: Cardiometabolic index; *LAP*: Lipid accumulation product; *VAI*: Visceral adiposity index; *AVI*: Abdominal volume index; *SHBG*: Sex hormone-binding globulin; *FSH*: Follicle stimulating hormone; *LH*: Luteinizing hormone; *E2*: Estradiol; *TT*: Testosterone; *HOMA*: Homeostatic model assessment. *P* < 0.05 was accepted statistically significant.
